# The Clinical Prediction Value of the Ubiquitination Model Reflecting the Microenvironment Infiltration and Drug Sensitivity in Breast Cancer

**DOI:** 10.7150/jca.101525

**Published:** 2025-01-01

**Authors:** Hai-Yan Ma, Jun-Ming Cao, Yuan-Yuan Zhang, Jin-Shuo Yang, Xin Wang, Yue Yu

**Affiliations:** 1 The First Department of Breast Cancer, Tianjin Medical University Cancer Institute and Hospital, National Clinical Research Center for Cancer, Tianjin 300060, China.; 2 Key Laboratory of Breast Cancer Prevention and Therapy, Tianjin Medical University, Ministry of Education, Tianjin 300060, China.; 3 Key Laboratory of Cancer Prevention and Therapy, Tianjin 300060, China.; 4 Tianjin Clinical Research Center for Cancer, Tianjin 300060, China.

**Keywords:** Breast cancer, ubiquitin, ubiquitin-related model, immune infiltration, drug sensitivity.

## Abstract

The ubiquitin-proteasome system influences cancer progression through multiple mechanisms. Due to the extensive proteasomal modifications observed in cancer tissues, ubiquitination is closely related to various biological functions with cancer. However, the roles of ubiquitin-related genes (UbRGs) in breast cancer (BC) have not been thoroughly investigated. In this study, we retrieved 763 reliable UbRGs and identified a potential prognostic signature for breast cancer patients. Additionally, we analyzed eight overall survival-associated UbRGs using univariate Cox proportional hazard regression in the Cancer Genome Atlas (TCGA) database. Subsequently, we used Lasso-Cox risk regression analysis to generate prognostic characteristics of UbRGs associated with overall survival (OS), validated in an external cohort (GSE158309). Next, we compared differences in tumor microenvironment and drug sensitivity between subgroups, describing the potential impact of UbRGs on the landscape of the tumor immune microenvironment and their predictive significance for therapeutic resistance to different strategies. Furthermore, a nomogram model containing eight genes, histology, subtype, T status, N status, and the American Joint Committee on Cancer (AJCC) stage was constructed. Finally, *in vitro* and *in vivo* experiments validated the effects of FBXL6 and PDZRN3 on breast cancer development. In conclusion, we demonstrate that ubiquitin-related genes are closely associated with breast cancer prognosis, immune environment, and drug sensitivity. Our results offer a new insight into breast cancer treatment.

## 1. Introduction

Breast cancer became the most prevalent cancer in the world in 2020 and was the fifth leading cause of cancer mortality worldwide^1^. Early-stage breast cancer patients generally have a better prognosis. However, advanced-stage breast cancer can still have a poor prognosis due to its varying heterogeneity^2^. Breast cancer has a complex pathogenesis that includes gene mutations, immune abnormalities, dysregulation of apoptosis, increased cell proliferation, invasion, and metastasis^3^. Targeted therapy has become a prominent area of research in breast cancer^4^. Therefore, we need to develop novel biomarkers for breast cancer detection and identify new therapeutic approaches to improve the prognosis of metastatic breast cancer^5^-^7^. Consequently, a new biomarker is still needed to identify such BC patients.

Ubiquitination is a common post-translational modification that regulates protein stability and degradation. It typically occurs in enzyme-dependent reactions and can be reversiblel^8^. Ubiquitination involves three main types of enzymes: ubiquitin-activating enzymes (E1s), ubiquitin-conjugating enzymes (E2s), and ubiquitin-protein ligases (E3s)^9^. These UbRGs form a complex network for modifying protein substrates^10^. UbRGs regulate many biological processes, including protein degradation and the cell cycle^11^. Numerous tumor-associated proteins undergo ubiquitination and are degraded in a proteasome-dependent manner^12^. Some ubiquitin-associated proteins interact with cancer-associated proteins as one of the critical mechanisms of breast cancer pathogenesis. It has been shown that RNF126 makes triple-negative breast cancer patients more sensitive to radiation by controlling DNA damage repair through the ATR-CHK1 pathway^13^. Thus, dysfunction in ubiquitination has the potential to promote the development of breast cancer^14^. Yang *et al.* selected four ubiquitination genes associated with breast cancer prognosis but did not perform analyses related to immune and drug susceptibility^15^. Zhao *et al.* revealed that UbRGs might interact with the immune phenotype of TNBC, but not in patients with whole breast cancer^16^. Thus, there remains a need to further reveal the predictive value of the UbRGs in breast cancer.

In this study, we constructed a reliable signature derived from UbRGs and systematically evaluated its role in the prognosis of BC patients. We revealed potential implications among TME features. We also explored its response to endocrine therapy, chemotherapy, and targeted therapy. Then, a monogram graph was created that combined risk scores with other clinical indicators to predict the survival probability of BC patients. Finally, the effects of FBXL6 and PDZRN3 on breast carcinogenesis were experimentally verified. Our analysis suggests that UbRGs may be useful as predictive biomarkers for breast cancer and are crucial to the disease's development.

## 2. Materials and Methods

### 2.1 Data acquisition and collection of UbRGs

**Figure 1:**
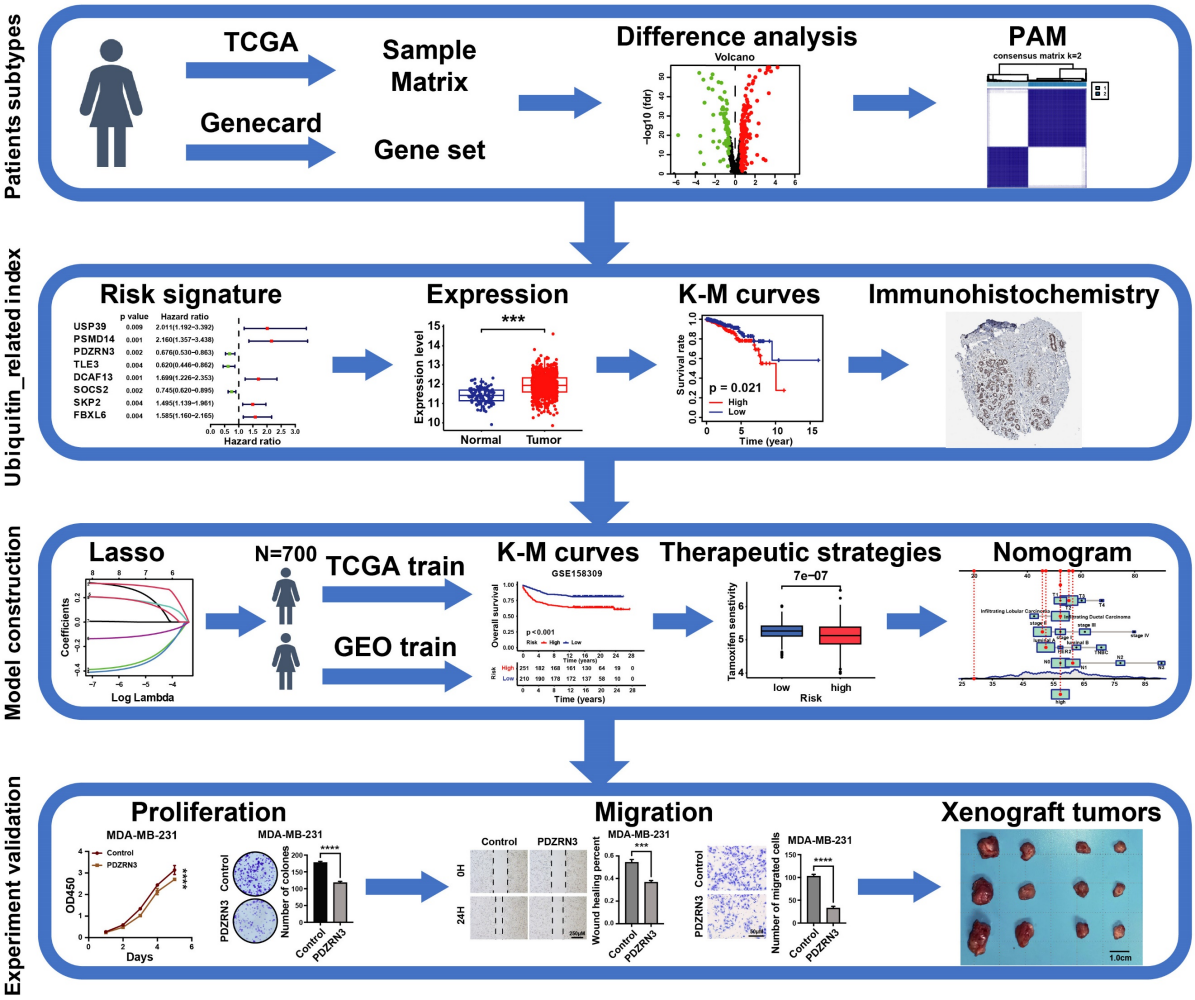
The flow chart of the study.

The workflow is shown in Figure 1. We collected 763 ubiquitin-related genes from the iUUCD 2.0 database for our study. The gene expression profiles and corresponding clinical data for breast cancer were obtained from TCGA (a training cohort) and GEO (an external validation cohort). After excluding patients without clinicopathological data or OS time information, we obtained 112 normal breast samples, 700 breast cancer samples from TCGA-BRCA, and 460 breast cancer patients from GSE158309.

### 2.2 Consensus clustering analysis

We used a method called "PAM" to identify patient subtypes based on the expression levels of UbRGs in the TCGA-BRCA dataset. The number of clusters was determined in the TCGA-BRCA cohort using the R package "ConsensusClusterPlus", and the process was repeated 1,000 times to ensure the stability of the classification^17^. We utilized Kaplan-Meier survival curves to compare overall survival across the different clusters in the TCGA dataset. We performed a three-dimensional principal component analysis (PCA) to visualize the differences in the distribution of subtypes. The “limma” R package was used to analyze differentially expressed genes between clusters. The screening criteria were |log2FC| ≥ 1 and an adjusted *p*-value < 0.05.

### 2.3 Establishment and evaluation of UbRGs prognostic signature

We used the "Survival" R package to conduct a one-way Cox regression analysis on the TCGA-BRCA dataset to identify possible prognostic genes associated with ubiquitination. Cox regression analysis with LASSO penalties was performed to identify potential signature genes^18^. Finally, multifactor Cox regression analysis was employed to determine the regression coefficients for each gene. According to the predictive model, the ubiquitin correlation index for each BC patient was determined using the following formula:



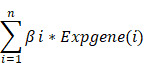



*n* stands for the number of genes in the predictive model, *β* represents the regression coefficient, and *Expgene(i)* denotes the expression level of each gene. To explore the importance of each essential gene, we examined the expression level of those genes in the TCGA-BRCA database. The KM analyzed the relationship between each gene and breast cancer prognosis. We divided the samples into low-risk and high-risk groups based on median risk scores. KM analyses of high-risk and low-risk groups of BC patients were performed in the TCGA-BRCA and GEO datasets for OS to assess the reliability of the prognostic impact of ubiquitin-associated signatures on breast cancer patients. The association between risk scores and survival status is presented in a visualization through bar charts. This study examined the OS of two groups of breast cancer patients with different estrogen receptor (ER) status, AJCC stages, T status, and N status. This was done to show that ubiquitin-related signatures affect the OS of BC patients in different subgroups. We also applied GSEA analysis to evaluate the clinical value of these subtypes.

### 2.4 Signaling pathways and cellular processes are affected by ubiquitin-related signatures

We analyzed differential genes in the two groups. We found differences in immune cell infiltration and immune function using the "GSEABase" software package for single-sample gene set enrichment analysis (ssGSEA). The CIBERSORT deconvolution technique is used to quantify the abundance of 22 tumor immune-infiltrating cell types in BC samples with high and low URI^19^. The analysis of potential pathways associated with DEG was enriched through Gene Ontology (GO) and Kyoto Encyclopedia of Genes and Genomes (KEGG) analyses using the "DOSE” and "org.Hs.eg.db" R packages.

### 2.5 Drug sensitivity analysis

The “prophetic” R package predicted the difference in drug sensitivity between the two groups^20^. The guidelines recommend using tamoxifen, fulvestrant, cyclophosphamide, cisplatin, paclitaxel, epirubicin, gefitinib, and lapatinib to treat breast cancer^21^. The differences in IC50 between the two drugs were analyzed using the original meaning while ensuring clarity.

### 2.6 Construction of calibration curves and nomograms

Based on these results, a ubiquitination-related clinicopathological nomogram model was developed using the "replot" and "rms" R packages. These packages combine URIs with age, T-status, N-status, histology, AJCC stage, and subtype in the training set. We plotted calibration curves and DCA for breast cancer patients to evaluate the predictive efficacy of the model.

### 2.7 Cell lines and cell culture

MCF10A, MDA-MB-231, and CAL51 cell lines were purchased from the Cell Bank of the Chinese Academy of Sciences (Shanghai, China). A specialised culture medium was used for MCF10A cells (Procell, China); DMEM medium (Gibco, USA) was used to culture MCF7 and MDA-MB-231 cells; and 1640 medium (Gibco, USA) was used to culture CAL51 cells. 1% penicillin/streptomycin solution (Gibco) and 10% fetal bovine serum (Gibco) were added to all media. All cell lines were cultured in a suitable incubator.

### 2.8 Cell transfection

The small interfering RNA of FBXL6, short hairpin RNA of FBXL6, Full-length cDNA of human PDZRN3, and their control plasmids were purchased from Riobo (Anhui, China). Stable down expression of FBXL6, overexpression of PDZRN3, and their control cell groups were established by lentivirus infection in MDA-MB-231 cells. Cells were transfected with siFBXL6-1 and siFBXL6-2 using the transfection reagent Lipofectamine®2000 (Invitrogen). Further experiments were performed on the transfected cells after 48 hours. The western blot analysis was performed to verify the knockdown and overexpression efficiency.

### 2.9 Western blot analysis

The cells were lysed with RIPA buffer (Solarbio, Beijing) containing one mM PMSF. The protein concentration was measured using the BCA Protein Assay Kit (Thermo Fisher Scientific). Proteins were separated using SDS-PAGE and transferred to a polyvinylidene difluoride membrane (Millipore, Bedford, MA, USA). The PVDF membranes were blocked using milk and then incubated with the corresponding primary antibodies overnight at 4°C. The next day, they were incubated with secondary antibodies for one hour at room temperature. The blots were developed using an enhanced chemiluminescence (ECL) reagent (Millipore). The antibody information used in this research is listed in Table S1.

### 2.10 RNA isolation and quantitative real-time PCR analysis

Total RNA from the cultured cells was extracted using the SPARKeasy Animal Tissue/Cell RNA Kit (SparkJade, China). A NanoDrop 3000 spectrophotometer (Thermo Scientific, USA) was used to measure the concentration and purity of RNA. Subsequently, the RNA was reverse-transcribed into cDNA for further experiments. TransStart SYBR Green qPCR SuperMix (TransGen, China) was used to set up the reaction system. The QuantStudio 5 Flex real-time PCR system (Applied Biosystems, USA) was used for the quantitative qRT-PCR. GAPDH was used as an internal standard. All samples were run in triplicate. The specific sequences are listed in Table S2.

### 2.12 Cell proliferation and colony-forming assay

For the Cell Counting Kit-8 assay, 2000 cells per well were plated into a 96-well plate and cultured in an incubator. Then, 100 µl of CCK8 solution was added to each well at a fixed daily time from the second to the fifth day. After an additional 2 hours of incubation, the cells in each well were measured at 450 nm (OD450).

For the colony-formation assay, 500 cells were inoculated into 6-well plates and cultured for three weeks. After that, the cells were fixed with cell fixative for 30 min and stained with 0.2% crystal violet for 15 minutes. The stained cells were washed with PBS three times and photographed under a microscope to count the colonies.

### 2.13 Cell invasion and migration assay

Transwell assays were used to investigate the ability of cells to invade and migrate. A transwell filter (Corning) was placed into a 24-well plate, culture medium and cell suspension in 20% FBS was added to the upper chamber, and serum-free culture medium was added to the lower chamber to detect migration ability. An additional 100 μl of Matrigel was added to the upper chamber to detect invasion ability. The cells in the upper chamber were removed after being incubated at 37°C for a specific duration. The cells in the lower chamber were then fixed with 4% paraformaldehyde, stained with 0.2% crystal violet solution, and subsequently photographed.

For the wound healing/scratch assay, cells were seeded into 6-well plates. After 24 hours, three vertical scratches were made in each well using a 20-μl sterile pipette tip to create a wound for assessment of cell migration. They were washed twice with PBS and incubated in a serum-free medium. Two randomly selected fields of view were photographed at 0H, 24H, and 48H after the scratches were produced. Distances were measured perpendicular to the edge of the scratch and analyzed using Adobe Photoshop software.

### 2.14 Xenograft and immunohistochemistry assays

1 × 107 cells were subcutaneously inoculated in female NOD/SCID/IL2 receptor γ null (NSG) mice. The tumor's size was measured every five days, and the tumor volume was calculated according to the formula [V = (L × W)2/2]. After thirty days, the animals were put down, and measurements were taken of the tumor's size and weight. The Animal Ethics Committee has authorized all animal research, and all researchers adhere to animal welfare rules.

The breast cancer tissues of mice were collected and fixed in 4% paraformaldehyde at 4 °C overnight. The tissue was then embedded, and the wax block was cut into thin slices (4 µm) using a microtome. The sections were dewaxed, and the first antibody was incubated overnight at 4 °C by retrieving the antigen in sodium citrate buffer. Incubate the secondary antibody the next day, then conduct DAB color development and hematoxylin staining. Dehydrate the samples and finally seal the film. The microscope camera was used to obtain images, and each slice was scored using immunohistochemistry. Table S1 lists the antibody information utilized in this study.

### 2.15 Statistical analysis

A student's t-test was used to assess the statistical significance of differences between the two data groups. A one-way ANOVA was used to determine the importance of differences between the study's three or more experimental groups. SPSS version 26.0 (IBM Corp, USA) was used for statistical analysis, and the GraphPad Prism software version 7.0.0 (GraphPad Inc, US) was used for plotting.

## 3. Results

### 3.1 The landscape and clustering analysis of UbRGs in breast cancer

**Figure 2:**
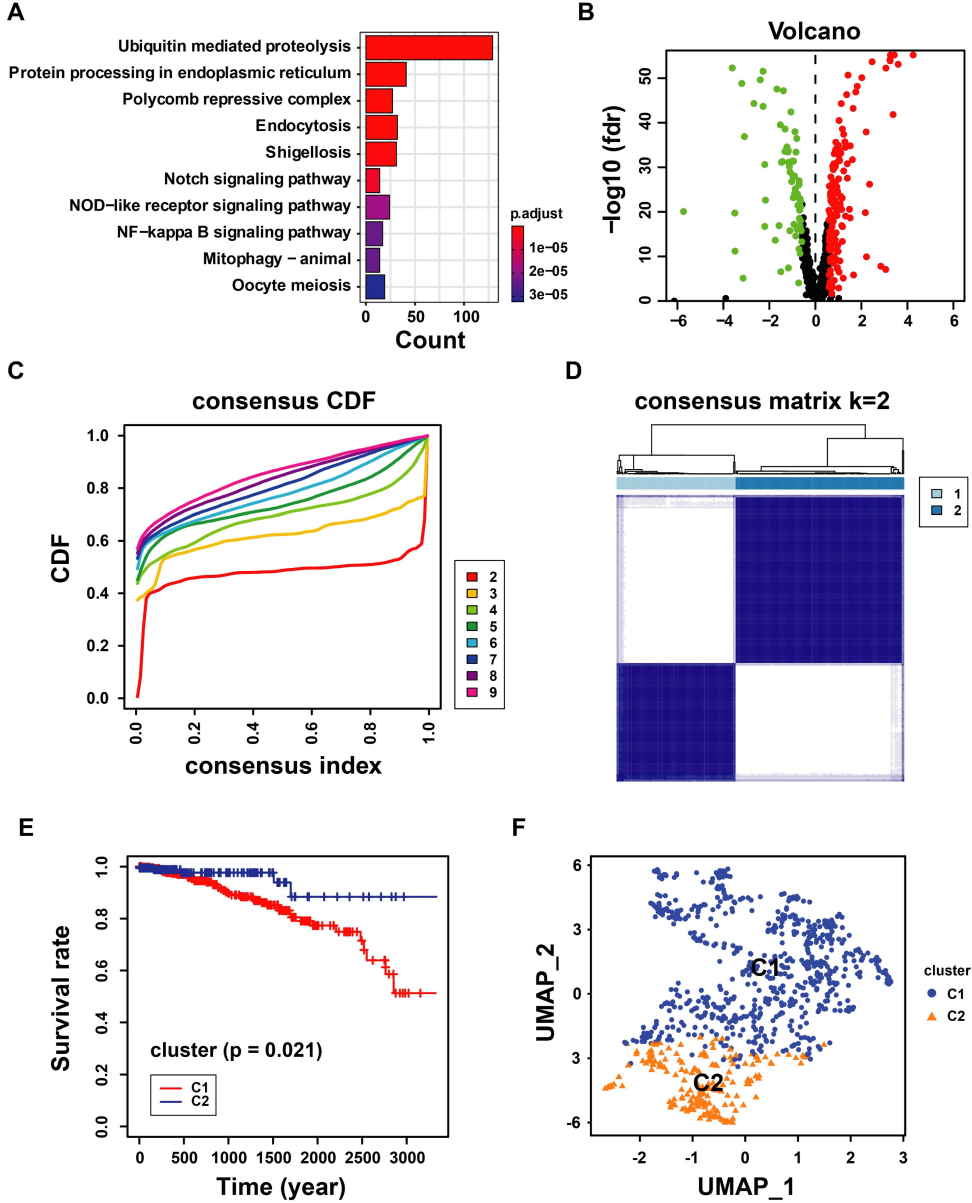
** The landscape and consensus clustering of UbRGs in BRCA. (A)** Bar plot of the Gene Ontology enrichment analysis of ubiquitin genes; **(B)** Volcano plot exhibiting 225 DEGs among UbRGs; **(C)** Consensus clustering cumulative distribution function (CDF) for k = 2-9; **(D)** Consensus clustering matrix for k = 2; **(E)** Kaplan-Meier analysis of overall survival for 622 patients with thyroid carcinoma from TCGA database; **(F)** Principal component analysis of the total RNA expression profile from TCGA database.

The ubiquitin-proteasome system is the signature pathway for degrading proteins involved in the breast cancer process^22^. We analyzed 763 UbRGs from the TCGA and GEO datasets to characterize the role of those genes in breast cancer patients. Our study flow chart is illustrated in Figure 1. The GO enrichment analysis showed that these UbRGs involve biological processes related to ubiquitinating proteins (Figure 2A). We found that 225 UbRGs were differentially expressed in breast cancer, of which 145 UbRGs were enhanced, and 70 UbRGs were attenuated (Figure 2B).

We identified two subgroups through a consistent clustering analysis of TCGA-BRCA patients, examining the various degrees of UbRG expression. According to the cumulative distribution function (CDF) curve, the best clustering was obtained when k was 2 (Figure 2C). When the K value was set to 2, TCGA-BCRA database patients were categorized into two subtypes: cluster 1 (n = 622) and cluster 2 (n = 190; Figure 2D). A survival analysis revealed that the OS time in cluster 1 was shorter than in cluster 2 (p < 0.05; Figure 2E). We performed a principal component analysis to prove whether the two subtypes can be separated. The results indicated that the two samples were well separated from each other (Figure 2F). The results suggest that ubiquitination genes may differ among breast cancer patients.

### 3.2 Identification of Prognostic UbRGs in Breast Cancer Patients

**Figure 3:**
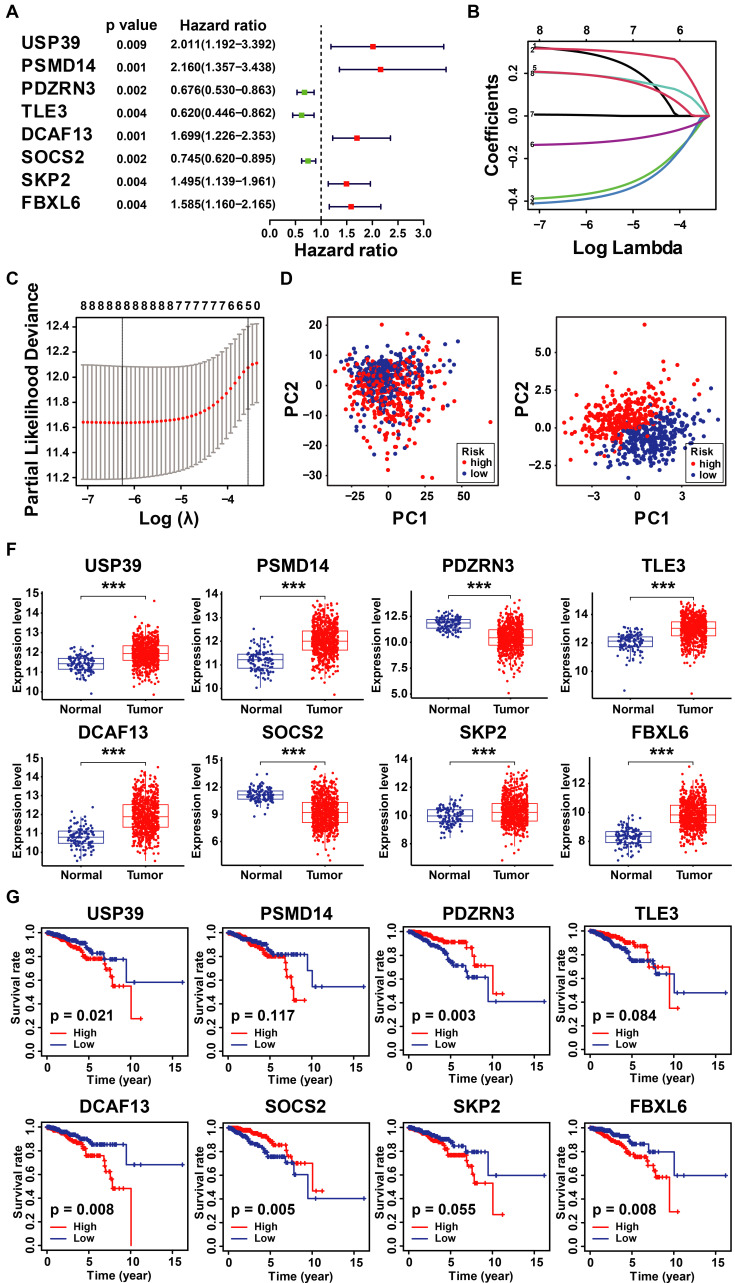
** Construction of ubiquitin-related signature in TCGA-BRCA cohort. (A)** Statistically significant (*p* < 0.01) UbRGs of the predictive model based on univariate Cox proportion hazards regression; **(B)** LASSO plot of 8 genes with ubiquitination LASSO model; **(C)** Partial likelihood deviance for the Lasso regression; **(D)** Principal component analysis of all the genes in the TGGA-BRCA cohort; **(E)** Principal component analysis of the gene-expression profiles in the TGGA-BRCA cohort; (F) The expression level of 8 UbRGs contained in the signature; **(G)** Kaplan-Meier **(K-M)** analyses of OS based on the expression level of 8 UbRGs. * *p* < 0.05; *** *p* < 0.001; ns means no significance.

**Figure 4:**
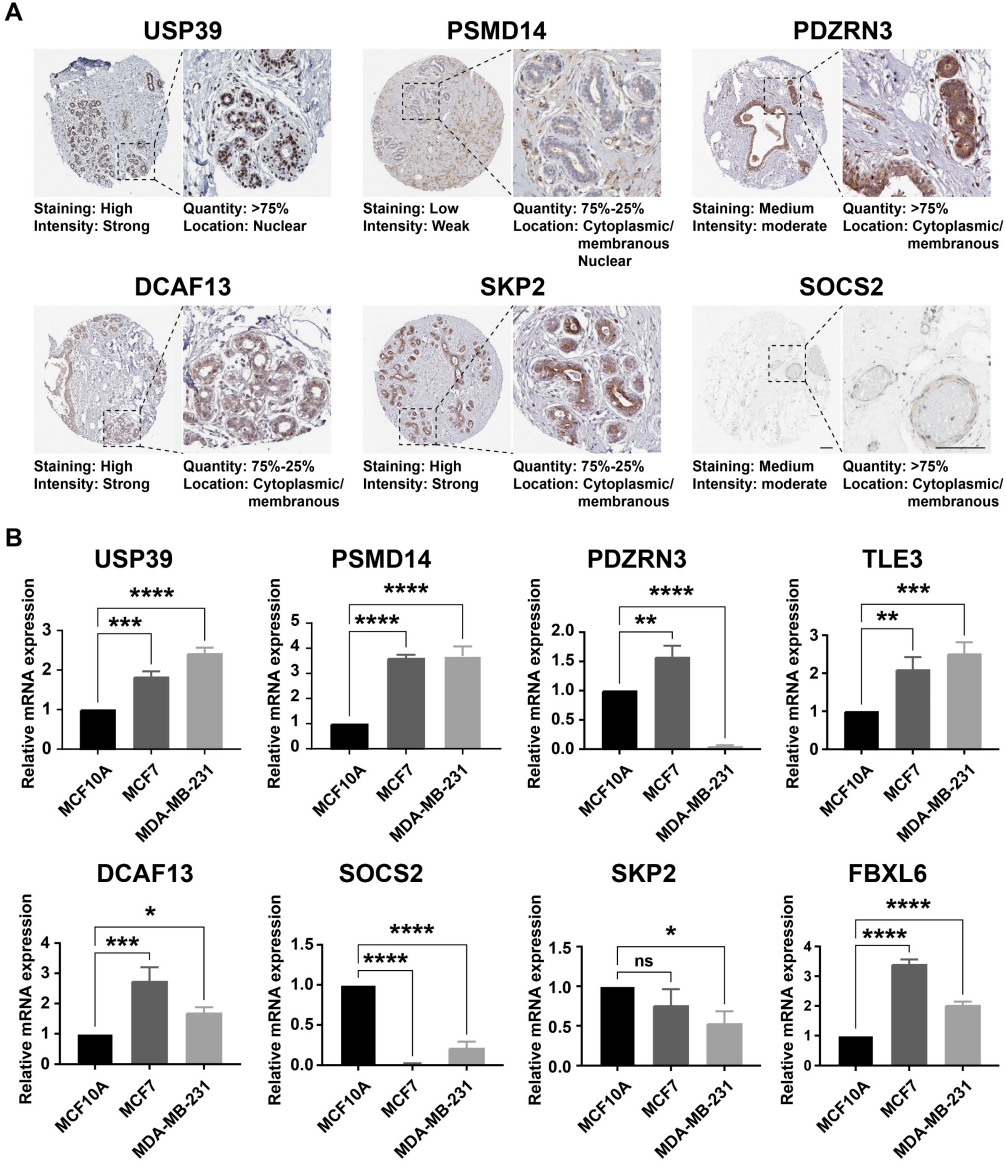
** The expression level of UbRGs. (A)** Analysis of the protein expression level of USP39, PSMID14, PDZRN3, DCF13, SKP2, and SOCS2 in breast cancer by the Human Protein Atlas (HPA) database; **(B)** The expression of USP39, PSMID14, PDZRN3, TLE3, DCF13, SKP2, SOCS2, and FBXL6 in indicated cells determined by RT-qPCR. * p < 0.05; ** *p* < 0.001; *** *p* < 0.001; **** *p* < 0.0001; ns means no significance.

Considering the prognostic significance of the different subtypes, we analyzed the key genes that differed in the TCGA-BRCA cohort based on the differential expression of UbRGs. Firstly, one-way Cox regression analysis showed that eight genes had the most significant difference in expression in breast cancer (*P*<0.001) (Figure 3A). We then performed Lasso-Cox regression analysis on candidate OS-related ubiquitin-related genes and identified the best UbRGs. Ultimately, based on the above eight genes, UbRGs signatures were established, which embodied USP39, PSMD14, PDZRN3, TLE3, DCAF13, SOCS2, SKP2, and FBXL6 (Figures 3B, C). As shown in Figures 3D and 3E, PCA analysis demonstrated that breast cancer patients could be well differentiated based on these eight specific genes. In addition, we aim to further demonstrate the different expression levels and independent predictive ability of each UbRGs in breast cancer. We used TCGA-BRCA data to show the expression level box line plots (Figure 3F) and the overall survival Kaplan-Meier curves (Figure 3G). We observed the expression of these genes in breast cancer tissues using the HPA database. The expression levels of UbRGs in MCF10A, MCF7, and MDA-MB-231cell lines were determined using RT-qPCR. The results of the analysis of data from the databases and RT-qPCR were consistent with one another. (Figure 4).

### 3.3 Construction and Validation of UbRGs Relevant Prognostic Signature for Breast Cancer Patients

**Figure 5:**
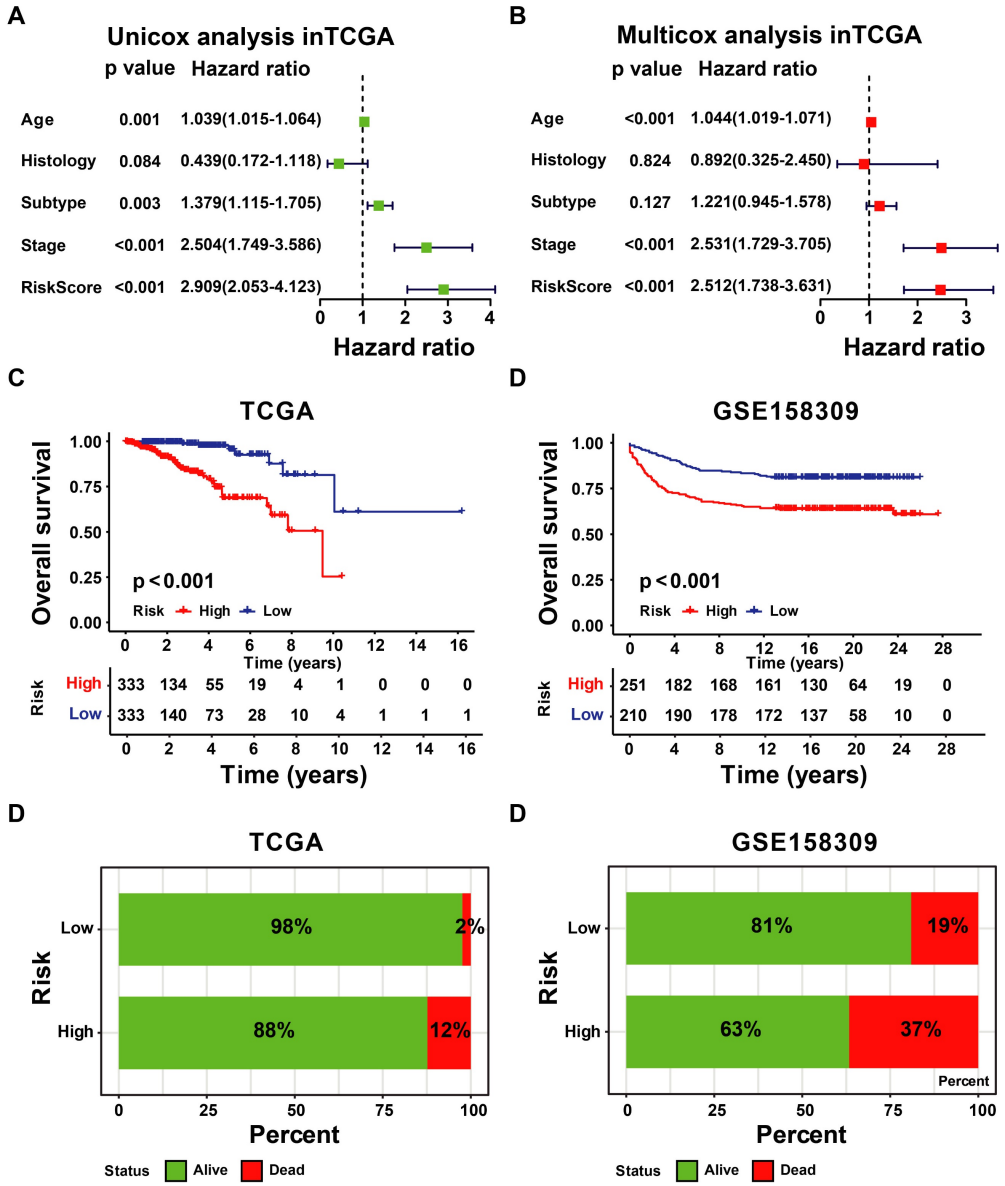
** Evaluation and validation of the utility of URI. (A)** The univariate Cox regression analysis in TCGA-BRCA cohort. **(B)** The multivariate Cox regression analysis in the GSE158309 cohort. **(C)** K-M analyses of OS between high- and low-URI groups in TCGA-BRCA cohort. **(D)** K-M analyses of OS between high- and low-URI groups in GSE158309 cohort. **(E-F)** The survival status distribution of the expression of the 8 UbRGs in patients in the training and testing sets.

**Figure 6:**
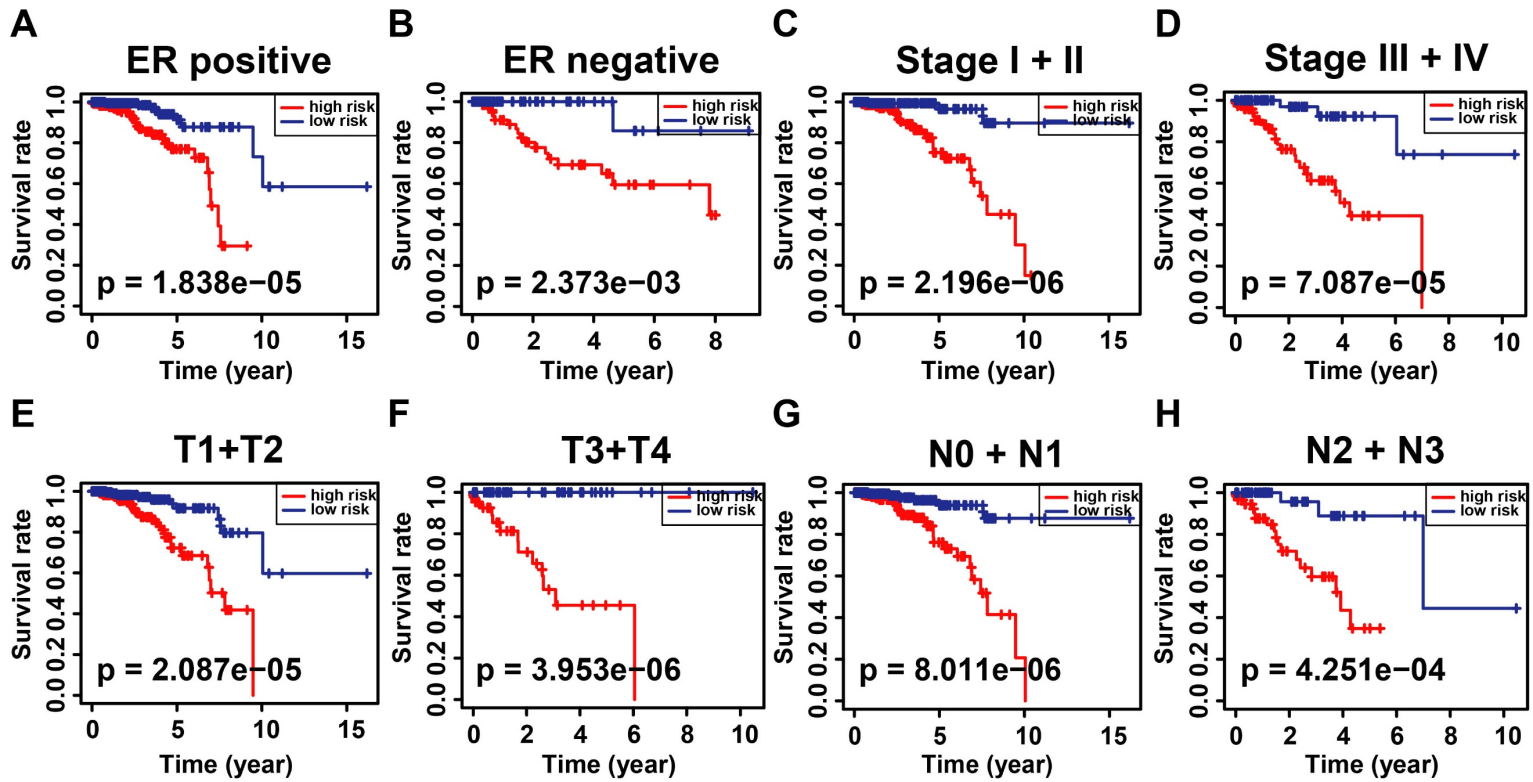
Kaplan-Meier analysis of overall survival in patients with thyroid carcinoma stratified by ER status **(A, B)**, stage **(C, D)**, T status **(E, F)**, and N status **(G, H)**.

According to the signature, the ubiquitin-related index (URI) of each patient was as follows: ubiquitin-related index (URI) = Expression of USP39 * 0.304120 + Expression of PSMD14 * 0.312790 - Expression of PDZRN3 * 0.373859 - Expression of TLE3 * 0.396868 + Expression of DCAF13* 0.197475 - Expression of SOCS2 * 0.130334 + Expression of SKP2* 0.004816 + Expression of FBXL6* 0.198389. In addition, we identified the risk score as an independent prognostic biomarker for breast cancer patients. The Age (*P* < 0.001, HR = 1.039, 95% CI = 1.015 - 1.064), Subtype (*P* = 0.003, HR = 1.379, 95% CI = 1.115 - 1.705), Stage (*P* < 0.001, HR = 2.504, 95% CI = 1.749 - 3.586), and Risk Score (*P* < 0.001, HR = 2.909, 95% CI = 2.053 - 4.123) were significantly correlated with the overall survival (Figure 5A). As shown in Figure 5B, Multifactor Cox regression analysis showed that the Age (*P* < 0.001, HR = 1.044, 95% CI = 1.019 - 1.071), Stage (*P* < 0.001, HR = 2.531, 95% CI = 1.729 - 3.705), and Risk Score (*P* < 0.001, HR = 2.512, 95% CI = 1.738 - 3.631) were identified as the independent prognostic factors in patients with breast cancer. URI values were calculated for each sample, and based on the mean value of URI, we categorized BC patients into high and low URI groups. We performed a K-M analysis to assess the predictive prognostic feasibility of URI. We found poorer OS in the high URI group than in the low URI group in the training set (Figure 5C) and the same result in the validation set (Figure 5D). As expected, the percentage of deaths among patients in the low-risk group in the TCGA database was lower than in the high-risk group (Figure 5E). In the GEO database, the percentage of deaths was also found to be different between groups. (Figure 5F). We also determined whether the risk score can predict clinical prognosis in breast cancer stratified by ER status (Figures 6A, B), stage (Figures 6C, D), tumor size (Figures 6E, F), and lymph node status (Figures 6G, H). We found that the risk score could predict outcomes better in the low stage than in the high stage (Figures 6C, D).

### 3.4 Integrated assessment of URI and clinical parameters in patients with breast cancer

**Figure 7:**
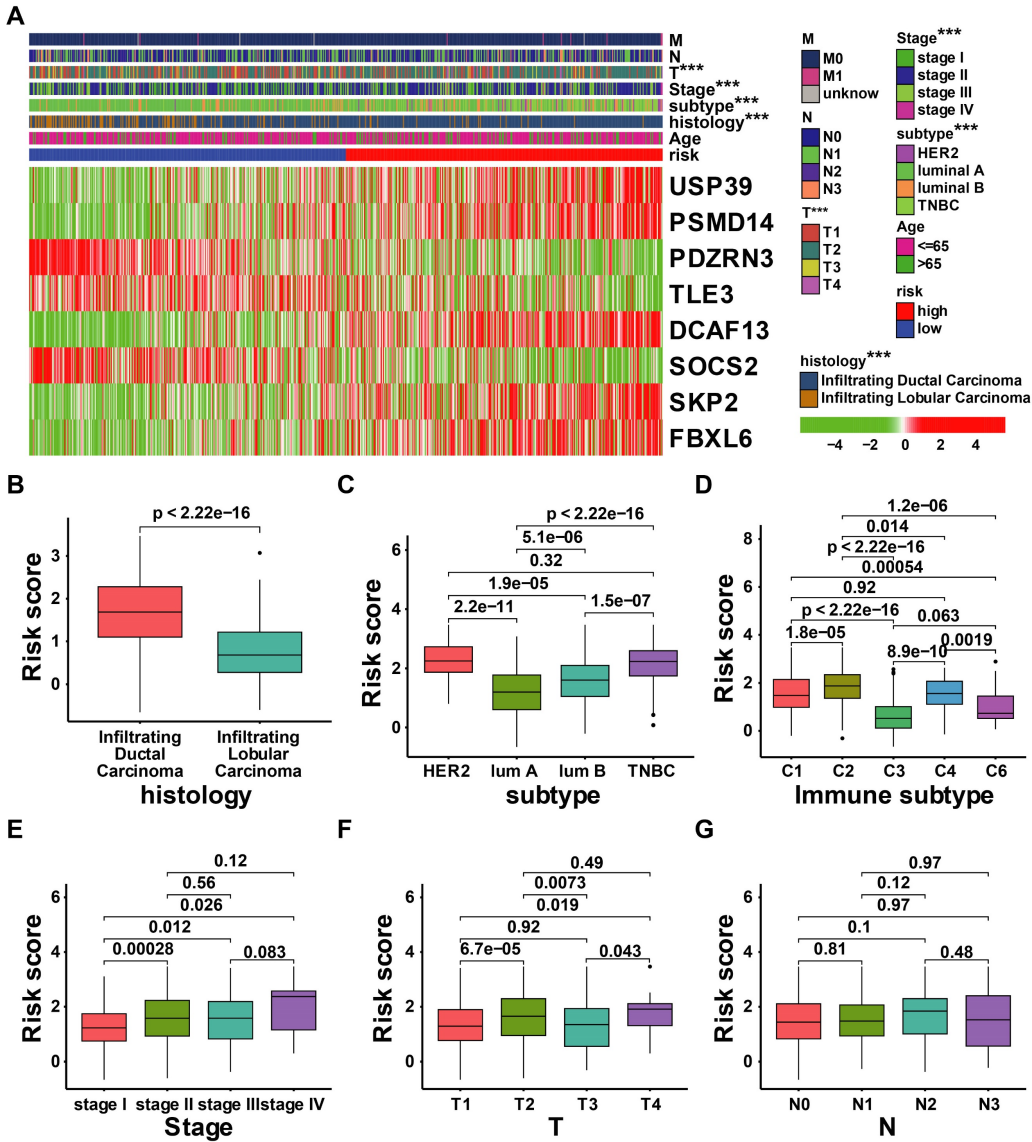
** Correlation analysis between the clinical features and risk score of patients with BRCA in the TCGA cohort. (A)** Correlation analysis between the risk signature and clinical characteristics in TCGA-BRCA cohort; **(B-G)** The comparison of risk scores between samples with different clinical characteristics, including histology **(B)**, subtype **(C)**, immune subtype**(D)**, stage **(E)**, T status **(F)**, and N status **(G)**. *** *p* < 0.001.

There have been many identified prognostic factors associated with cancer, including age, subtype, histology, AJCC stage, and TNM status. We further examined the relationship between URI and various clinical characteristics. As illustrated in Figure 7A, the risk score was significantly associated with T status, AJCC stage, subtype, and histology. Patients with high-risk scores exhibit adverse clinical features (Figures 7B-G).

### 3.5 The tumor microenvironment and drug sensitivity in patients with UbRGs relevant prognostic signature

**Figure 8:**
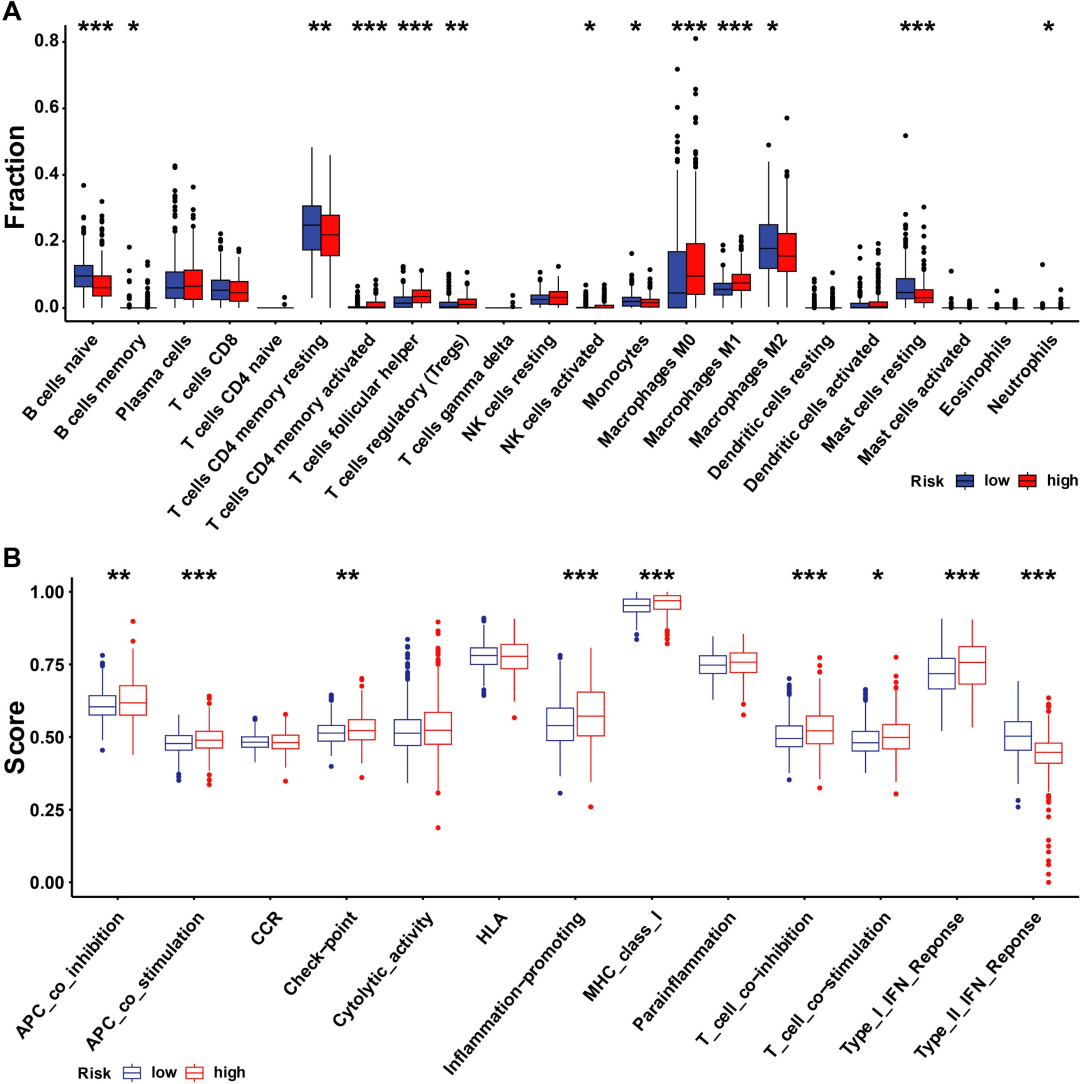
** Immune landscape and immune cells abundance between the high- and low-URI groups. (A)**The analysis of differences in immune cell infiltration between the two groups with ssGSEA; **(B)** The analysis of differences in immune functions between the two groups with ssGSEA. * p < 0.05; ** *p* < 0.001; *** *p* < 0.001.

We investigated whether there is a correlation between tumor microenvironment (TME) and URI using the R package. The high-risk group had high infiltration of regulatory T cells (Tregs), M0 macrophages, follicular helper T cells, active CD4 memory T cells, and M1 macrophages, while the high-risk group had high naive B cells, resting CD4 memory T cells, M2 macrophages, and resting mast cells (Figure 8A). Next, we examined differences in the expression patterns of immune-related genes in low-risk and high-risk patients (Figure 8B). Most immune-related cells were expressed at low levels in the high-risk group.

**Figure 9:**
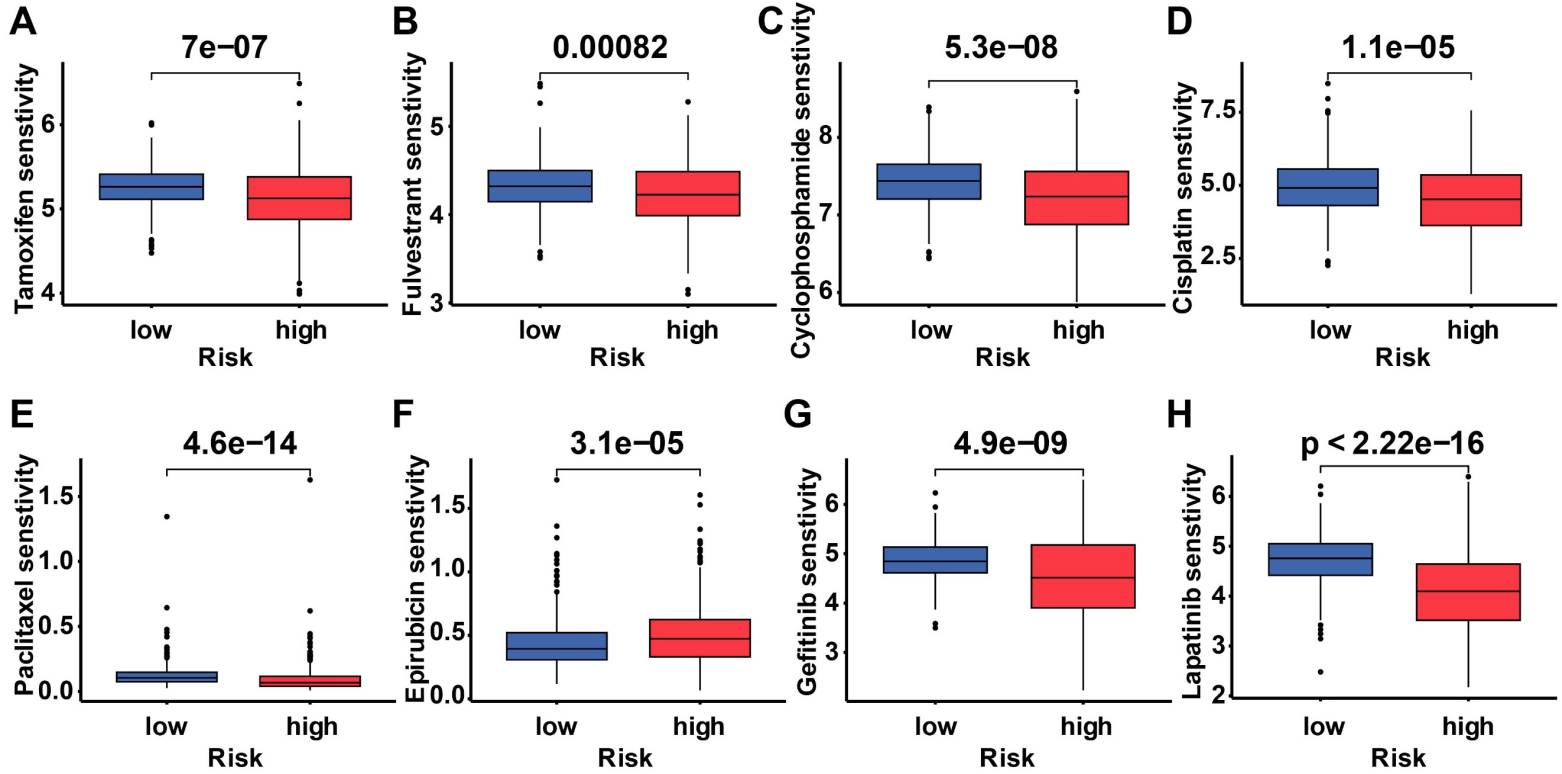
** Drug response of subgroups.** Prediction of the drug sensitivity (IC50) of **(A)** tamoxifen, **(B)** fulvestrant, **(C)** cyclophosphamide, **(D)** cisplatin, **(E)** paclitaxel, **(F)** epirubicin, **(G)** gefitinib, and **(H)** lapatinib. *p* < 0.05 is considered statistically significant.

To determine the difference in drug sensitivity between low-risk and high-risk categories, we correlated breast cancer patients' ubiquitination gene-related risk score with the IC50 values of chemotherapy, endocrine therapy, and targeted therapy. The IC50 values of epirubicin were significantly higher in the high-risk group, while the IC50 values of the other drugs were lower in this group (Figure 9).

### 3.6 Development and evaluation of ubiquitin-correlated clinicopathologic nomogram

**Figure 10:**
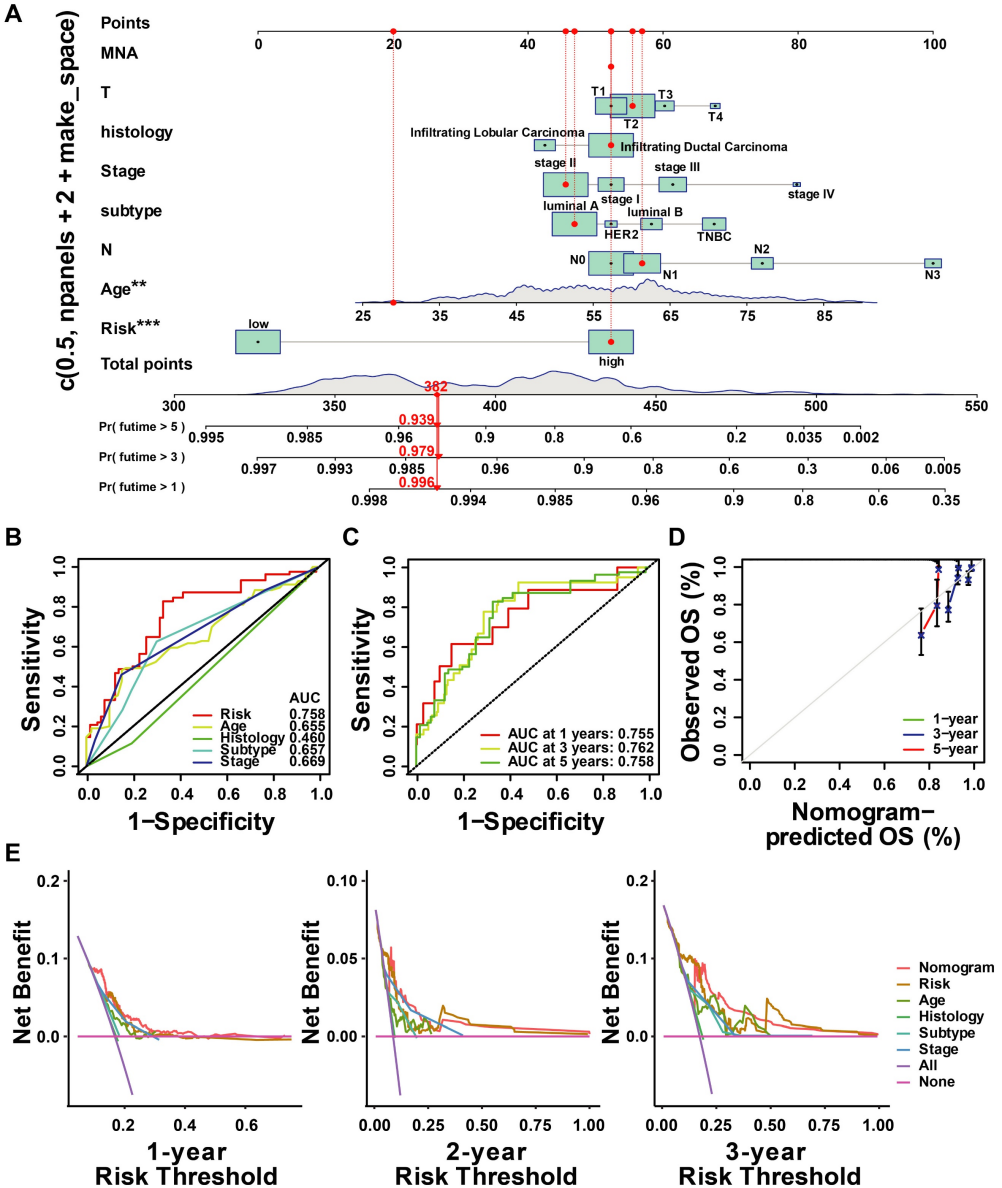
** Construction and assessment of nomogram. (A)**The prediction of nomogram in the TCGA-train dataset; **(B-C)** The multifactor AUC for 3-year survival; **(D)** Calibration curve to evaluate the consistency of predicted and actual OS; **(E)** Decision curve analysis (DCA) to assess the clinical decision-making benefits of the nomogram.

Based on the results above, a clinicopathologic nomogram was constructed, which included risk score, age, AJCC stage, histology type, and T/N stage, to predict the probability of OS of 1, 3, and 5 years in BC patients from the TCGA cohort (Figure 10A). ROC analysis showed the sensitivity and specificity of the ubiquitin-associated nomogram risk score to predict 1-, 3-, and 5-year survival (Figure 10C). We plotted calibration plots to confirm the column charts' predictive efficacy. We found that the ubiquitination gene-related column charts could better predict the prognosis of breast cancer patients (Figure 10D). DCA analysis also showed ubiquitin-related features were more sensitive and specific than clinicopathologic features (Figure 10E).

### 3.7 Downregulation of FBXL6 inhibited proliferation and promoted metastasis in breast cancer cells

**Figure 11:**
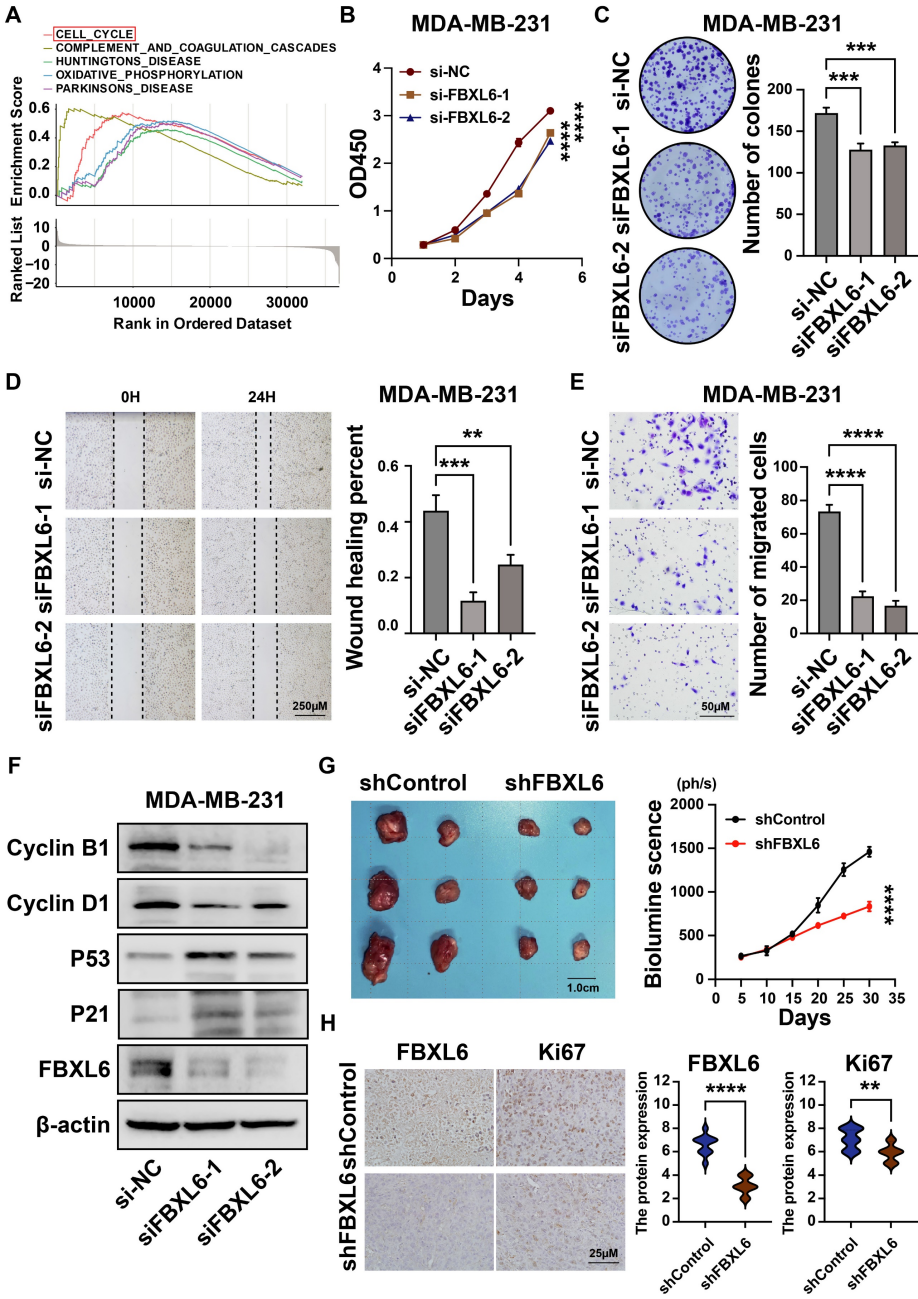
** Downregulation of FBXL6 inhibited the proliferation and migration of MDA-MB-231 breast cancer cells. (A)**Top five significantly enriched categories in the FBXL6-high group compared with its counterpart in TCGA; **(B)** The CCK-8 assay was performed to measure the proliferation capacity of MDA-MB-231 cells; **(C)** The colony formation assay and corresponding statistical analysis of MDA-MB-231 cells. The effect of FBXL6 on the migration of MDA-MB-231 cells determined by wound healing **(D)** and Transwell **(E)** assays; **(F)** Western blot analysis revealed that downregulation of FBXL6 inhibited the cell cycle progression in MDA-MB-231 cells; **(G)** Tumor growth of subcutaneous xenograft tumors comprising shFBXL6 or shCcontrol cells at the indicated times; H, FBXL6 and Ki67 protein expression levels in the xenograft tumors. ** *p* < 0.001; *** *p* < 0.001; **** *p* < 0.0001.

We found that FBXL6 was highly expressed in breast cancer tissues and negatively correlated with prognosis (Figures 3F-3G). Next, we analyzed the differential enrichment of biological signaling pathways between the FBXL6 expression groups using GSEA. The results showed that patients with high FBXL6 expression had a significant enrichment score in the cell cycle (NES = 1.90, NOM *p* = 0.004) (Figure 11A). We then downregulated the expression of FBXL6 in breast cancer cells by constructing a siRNA specific to FBXL6 and performed cellular function studies. The western blot was performed to verify the downregulation of FBXL6 expression (Figure 11F). The downregulation of FBXL6 inhibited proliferation and migration in breast cancer cells (Figures 11B-E). Furthermore, western blot analysis showed decreased cell cycle protein D1 expression and increased expression of p53, P21, cyclin B1, and cyclin D1 (Figure 11F). This suggests that the downregulation of FBXL6 inhibits cell cycle progression. Next, we performed functional experiments by reducing FBXL6 expression in CAL51 cells and obtained the same results (Figure S3). As expected, FBXL6 depletion inhibited cell proliferation and migration in CAL51 cells by blocking cell cycle transitions. Moreover, the MDA-MB-231 shFBXL6 cells and MDA-MB-231 shControl cells were implanted into the fat pad of NSG mice, and tumor growth was recorded at regular intervals. Down expression of FBXL6 in MDA-MB-231 cells significantly inhibited tumor growth *in vivo* (Figure 11G). IHC staining revealed that Ki-67 expression was down-regulated in tumors from MDA-MB-231 shFBXL6 mice compared to MDA-MB-231 shControl mice (Figure 11H).

### 3.8 PDZRN3 inhibited breast cancer cell growth, migration, and invasion

**Figure 12:**
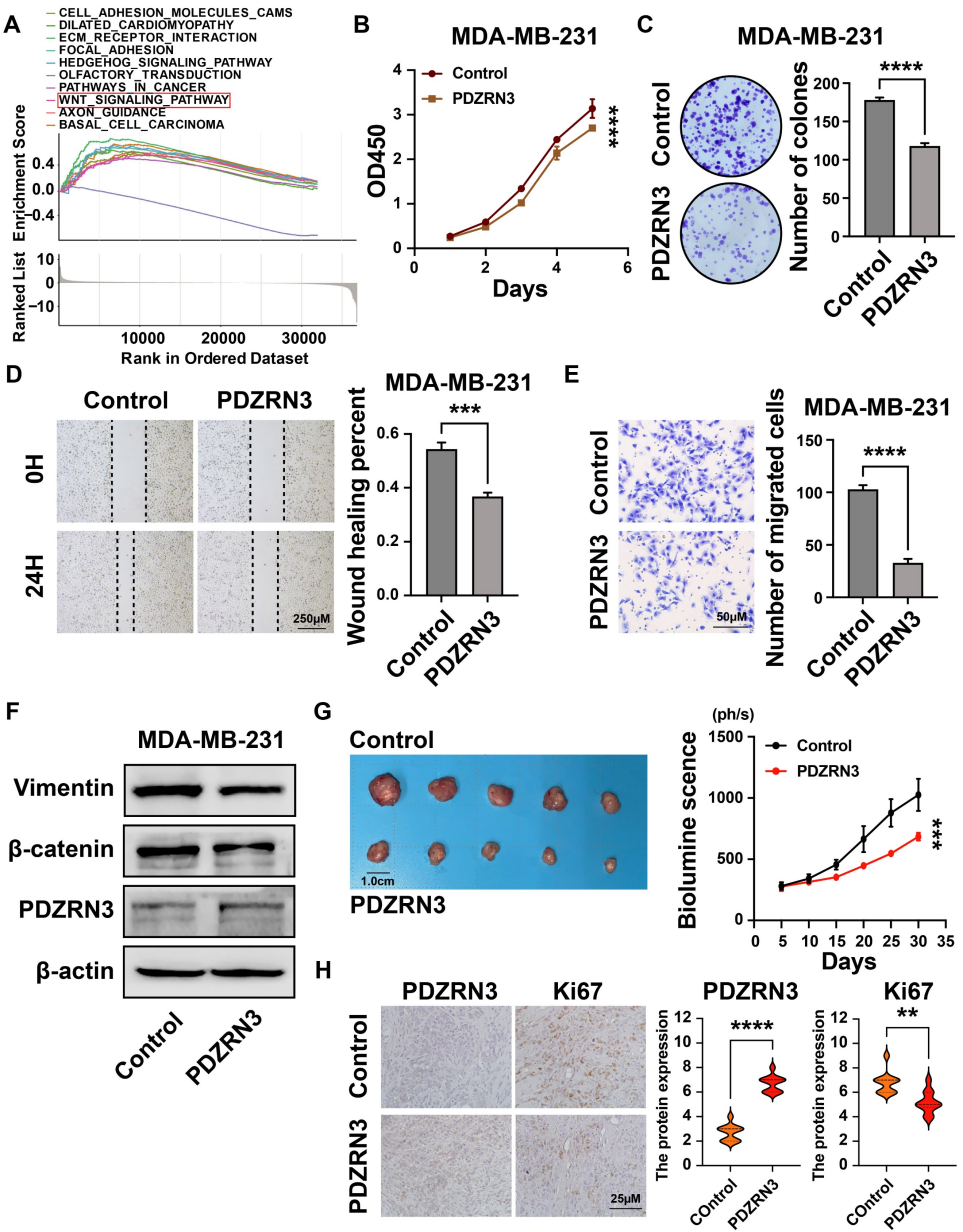
** PDZRN3 inhibited the proliferation and migration of MDA-MB-231 breast cancer cells. (A)**Top ten significantly enriched categories in the PDZRN3-high group compared with its counterpart in TCGA; **(B)** The CCK-8 assay was performed to measure the proliferation capacity of MDA-MB-231 and CAL51 cells; **(C)** The colony formation assay and corresponding statistical analysis of MDA-MB-231 and CAL51 cells; The effect of PDZRN3 on the migration of MDA-MB-231 and CAL51 cells determined by wound healing **(D)** and Transwell **(E)** assays; **(F)** Western blot analysis of the role of PDZRN3 in MDA-MB-231 and CAL51 cells; **(G)** Tumor growth of subcutaneous xenograft tumors comprising PDZRN3 or control cells at the indicated times. **(H)** PDZRN3 and Ki67 protein expression levels in the xenograft tumors. ** *p* < 0.001; *** *p* < 0.001; **** *p* < 0.0001.

Our results show that the expression of PDZRN3 in breast cancer is low, and its expression is related to the prognosis of patients (Figures 3F and 3G). The GSEA analysis revealed that the WNT signaling pathway had a significant enrichment score (NES = 1.688, NOM *p* = 0.007) (Figure 11A). We then verified this through cellular experiments. First, we transfected the PDZRN3 overexpression plasmid into the breast cancer cell line MDA-MB-231 to increase the expression of PDZRN3(Figure 12F). Functional experiments showed that the upregulation of PDZRN3 inhibited the proliferation and migration of MDA-MB-231 cells (Figures 12B-E). Finally, we used western blotting to validate that the insert of PDZRN3 exhibited decreased expression of β-catenin and vimentin (Figure 12F). We also performed functional experiments by upregulating PDZRN3 expression in CAL51 cells and obtained the same results (Figure S4). *In vivo*, the 231-Control and 231-PDZRN3 cells were injected subcutaneously. The results showed that over-expression of PDZRN3 significantly inhibited tumor growth (Figure 12G). IHC staining revealed that tumors from 231-PDZRN3 mice express a lower level of (Figure 12H).

## 4. Discussion

In the past decades, the incidence rate of breast cancer in many countries around the world has continued to rise. However, the 5-year survival rate for patients with advanced breast cancer remains low. Evidence from previous studies suggests that ubiquitin plays an integral role in the mechanism underlying breast cancer development^23^-^25^. Tumor classification studies based on ubiquitin correlation profiles emerge^26^, ^27^. By clarifying the role of ubiquitin-related genes in breast cancer heterogeneity, researchers can develop more effective treatment strategies for breast cancer. In the present study, we found that UbRGs are differentially expressed in breast cancer and that these differential genes can regulate cancer through various biological processes.

Univariate and multivariate Cox proportional hazards regression identified prognostic models in this study that were significantly associated with overall survival in cancer patients (*P* < 0.001). Eight genes were incorporated into this model, consisting of USP39, PSMD14, PDZRN3, TLE3, DCAF13, SOCS2, SKP2, and FBXL6. The UbRGs signature was validated in the test set (GSE158309). Of the eight UbRGs, USP39, PSMD14, DCAF13, SKP2, and FBXL6 are risk factors of BC, and PDZRN3, TLE3, and SOCS2 are protective factors of BC. We discussed these UBRG features. According to recent research, USP39 is significantly expressed in a broad spectrum of cancerous tumors. It has a role in several biological processes, including cell proliferation^28^, cell cycle progression^29^, cell migration and invasion^30^, cell apoptosis^31^, cell regulation^32^, and drug tolerance^33^. The results of our study indicated that USP39 is highly expressed in breast cancer and is associated with a poor prognosis. In several types of cancer, the oncogene PSMD14 has been found to encode deubiquitylation enzymes that function in the ubiquitin pathway^34^. Our study found that PSMD14 is highly expressed in BC tissues and acts as a risk factor for BC. DCAF13 is a novel substrate receptor for E3 ubiquitin-conjugating enzymes that regulate the progression of the cell cycle^35^. A previous study found that DCAF13 promotes the polyubiquitination of PERP, a protein downstream of the transcription of p53 and p63, thereby promoting breast cancer proliferation^36^. SKP2 leads to the degradation of tumor suppressor FOXO and CDK inhibitor P27, rendering these tumor suppressor genes inactive^37^. Our results also demonstrate that DCAF13 and SKP2 play essential roles in breast cancer prognosis. E3-FBXL6 degrades ETV6 through the ubiquitin-proteasome system and is involved in the growth and differentiation of cells^38^. A recent study showed that FBXL6 degrades P53 through polyubiquitination and proteasomal degradation, leading to the proliferation of colorectal cancer cells^39^. Our research results indicate that FBXL6 is highly expressed in breast cancer and promotes the progression of breast cancer through cell cycle regulation *in vivo*.

TLE3 is a transcriptional repressor of β-catenin and is vital in regulating the Wnt signaling pathway^40^. TLE3 also binds to FOXA1 and ER on ER target genes, playing a role in HR-positive breast cancer^41^. TLE3 is one of the critical signatures we use to predict the prognosis of breast cancer. PDRRN3 is a NUMB protein X family ligand member and contains the Ring type ubiquitin E3 ligase^42^. Li *et al.* demonstrated that the downregulation of PDZRN3 promotes EC cell metastasis and proliferation by activating the classical Wnt signaling pathway^43^. Our study found that PDZRN3 had Low expression in breast cancer and inhibited cell progression.

Recently, numerous findings have confirmed that the TME plays a crucial role in the development and progression of cancer^44^. In addition, tumor-infiltrating immune cells, a critical TME component, also promote tumor progression^45^, ^46^. To date, the association between ubiquitin and the overall TME infiltration characterizations and heterogeneity of breast cancer has not been comprehensively recognized. In our study, patients in the low-risk group exhibited higher levels of naive B cells, resting memory CD4 T cells, M2 macrophages, and resting mast cells. The high-risk group had a high infiltration of activated CD4 memory T cells, M0 Macrophages, follicular helper T cells, regulatory T cells (Tregs), and M1 Macrophages. In addition, UbRGs are associated with drug sensitivity in cancer therapy^27^. The patients in the high-risk group were more sensitive to endocrine therapeutics drugs (such as tamoxifen and fulvestrant) and chemotherapeutic drugs (such as cyclophosphamide, cisplatin, paclitaxel, and epirubicin). They targeted drugs such as Gefitinib and Lapatinib. Nevertheless, patients in the high-risk group exhibited higher resistance to epirubicin. In our study, prognostic characterization based on UbRGs shows excellent potential to assist in clinical treatment selection for BRCA patients.

In this study, we systematically analyzed the mapping of UbRGs in TCGA-BRCA patients and constructed prognostic characterization URIs based on OS-related UbRGs. Nevertheless, our research still needs some improvement. Since most analyses used data from publicly available datasets and all samples were retrieved retrospectively, case selection bias may exist. In addition, more experiments are still needed to prove our findings.

## 5. Conclusion

In this study, we created a novel ubiquitin-related risk profile that effectively forecasts cancer prognosis. Moreover, we demonstrated variations in clinical parameters, immune landscapes, and drug sensitivities among risk groups. Then, we established a nomogram model that predicts the prognosis of breast cancer patients by combining eight ubiquitination-related genes with clinical factors. In addition, both *in vitro* and *in vivo* experiments have demonstrated that FBXL6 and PDZRN3 can influence the growth of breast cancer cells. Taken together, the eight-gene model may serve as a prognostic marker in future clinical practice.

## Supplementary Material

Supplementary figures and tables.

## Abbreviations

UbRGs: Ubiquitin-related genes
BC: Breast cancer
TCGA: The cancer genome atlas
GEO: Gene Expression Omnibus
OS: Overall survival
AJCC: American joint committee on cancer
E1s: Ubiquitin-activating enzymes
E2s: Ubiquitin-conjugating enzymes
E3s: Ubiquitin-protein ligases
IHC: Immunohistochemistry
PCA: Principal component analysis
ER: Estrogen receptor
GSEA: Gene set enrichment analysis
ssGSEA: Single-sample gene set enrichment analysis
GO: Gene ontology
KEGG: Kyoto encyclopedia of genes and genomes
ECL: Enhanced chemiluminescence
CDF: Cumulative distribution function
URI: Ubiquitin-related index
TME: Tumor microenvironment
