# Supplementary material for: The Clinical Prediction Value of the Ubiquitination Model Reflecting the Microenvironment Infiltration and Drug Sensitivity in Breast Cancer
Source: J Cancer. 2025 Jan 1;16(3):784–801. doi: 10.7150/jca.101525 (PMC11705054; doi:10.7150/jca.101525)
Supplement: Supplementary file 1 — Supplementary figures and tables. [file jcav16p0784s1.pdf]

Supplemental Figures and Tables for

The Clinical Prediction Value of the Ubiquitination Model Reflecting the Immune Traits in Breast Cancer

Figure S1

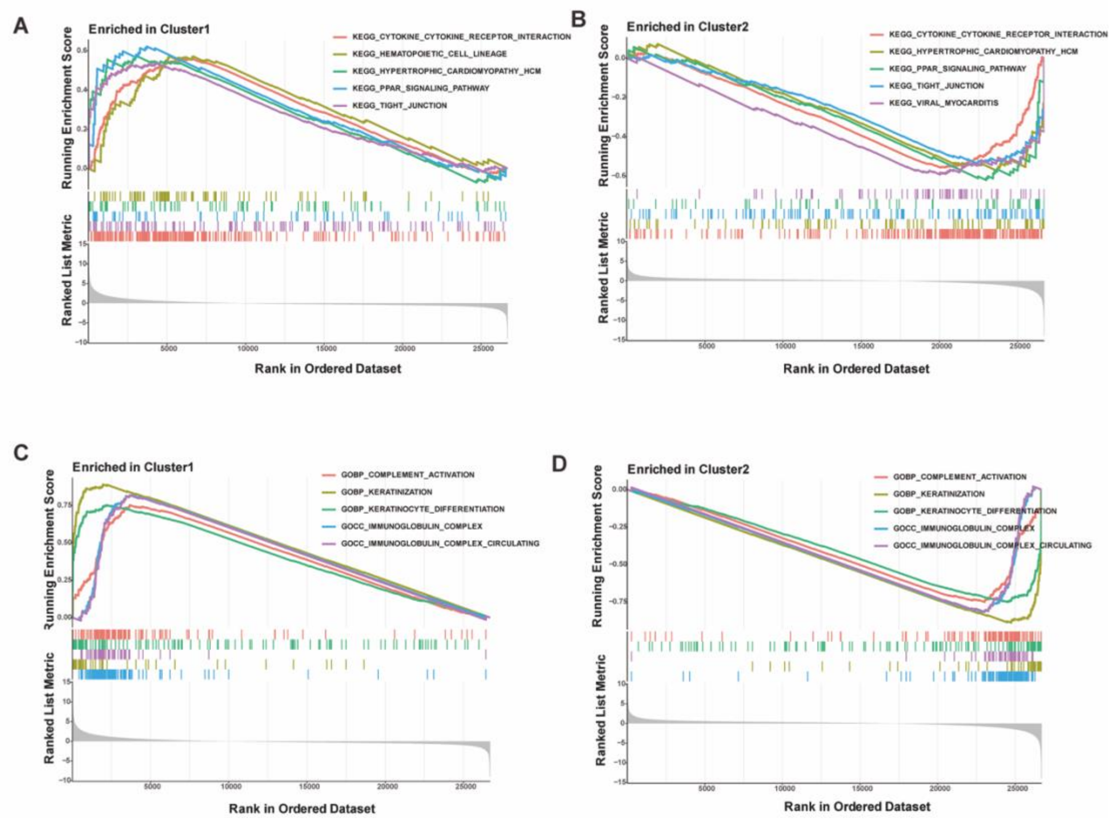

Figure S1 Gene Set Enrichment Analysis (GSEA) between the Cluster 1 and Cluster 2.

(A-D) Results of GSEA in TCGA-BRCA cohort.

Figure S2

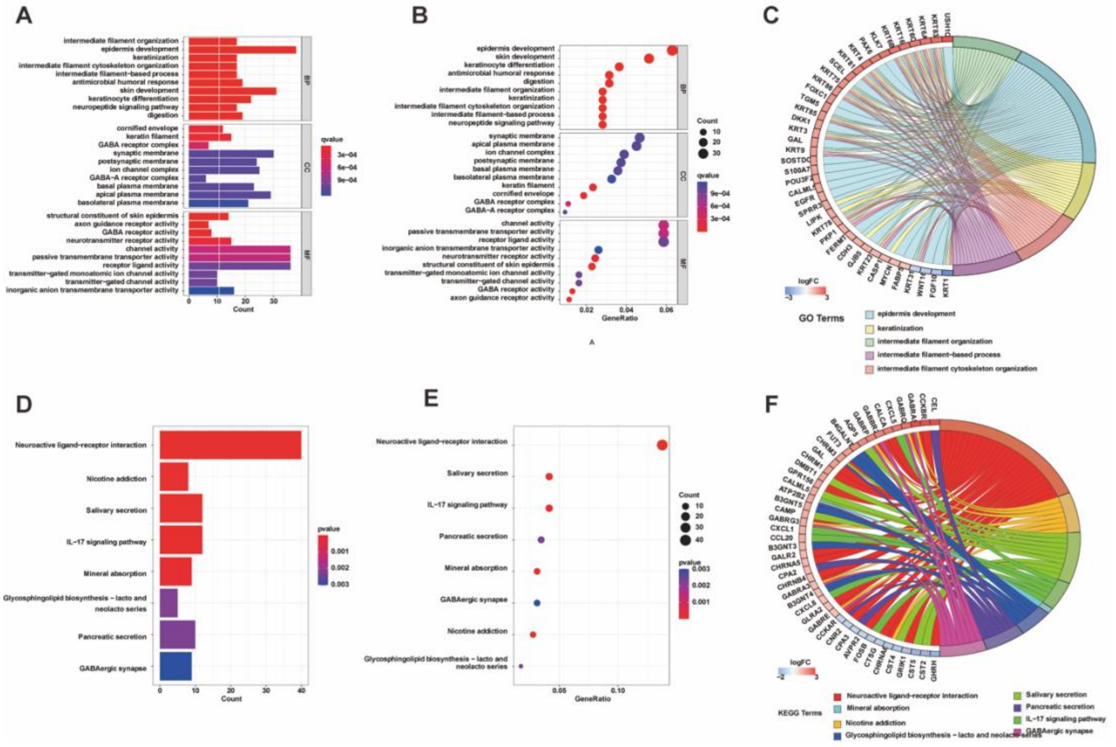

Figure S2 Differential signaling pathways and cellular processes between the high-risk subgroup and the low-risk subgroup

(A-C) Biological processes of differentially expressed genes between the high-risk subgroup and the low-risk subgroup. (D-F) KEGG pathways in which differentially expressed genes were distributed.

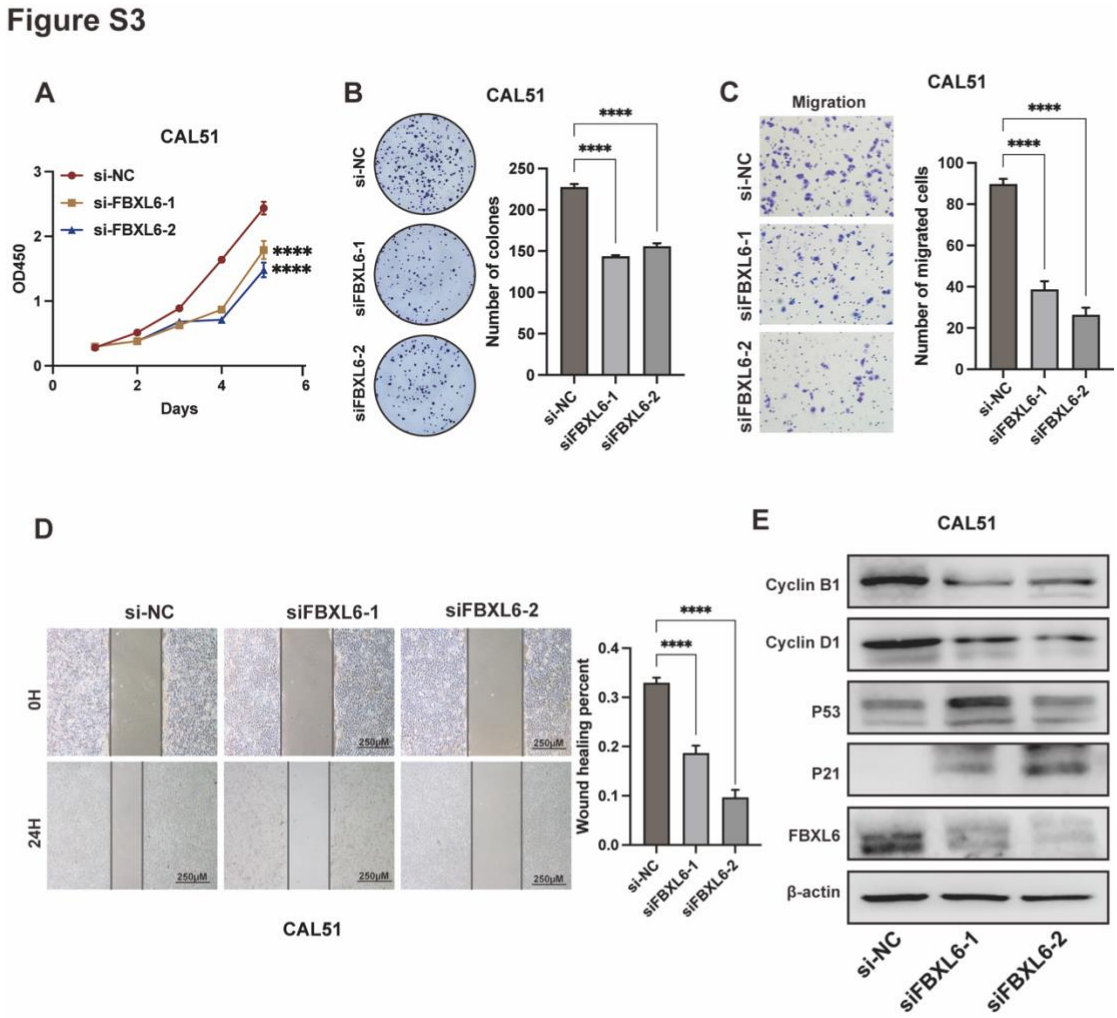

Figure S3 Downregulation of FBXL6 inhibited the proliferation and migration of CAL51 breast cancer cells.

(A) The CCK-8 assay was performed to measure the proliferation capacity of CAL51 cells; (B) The colony formation assay and corresponding statistical analysis of CAL51 cells; The effect of FBXL6 on the migration of CAL51 cells determined by transwell (C) and wound healing (D) assays; (E) Western blot analysis revealed that downregulation of FBXL6 inhibited the cell cycle progression in CAL51 cells.

Figure S4

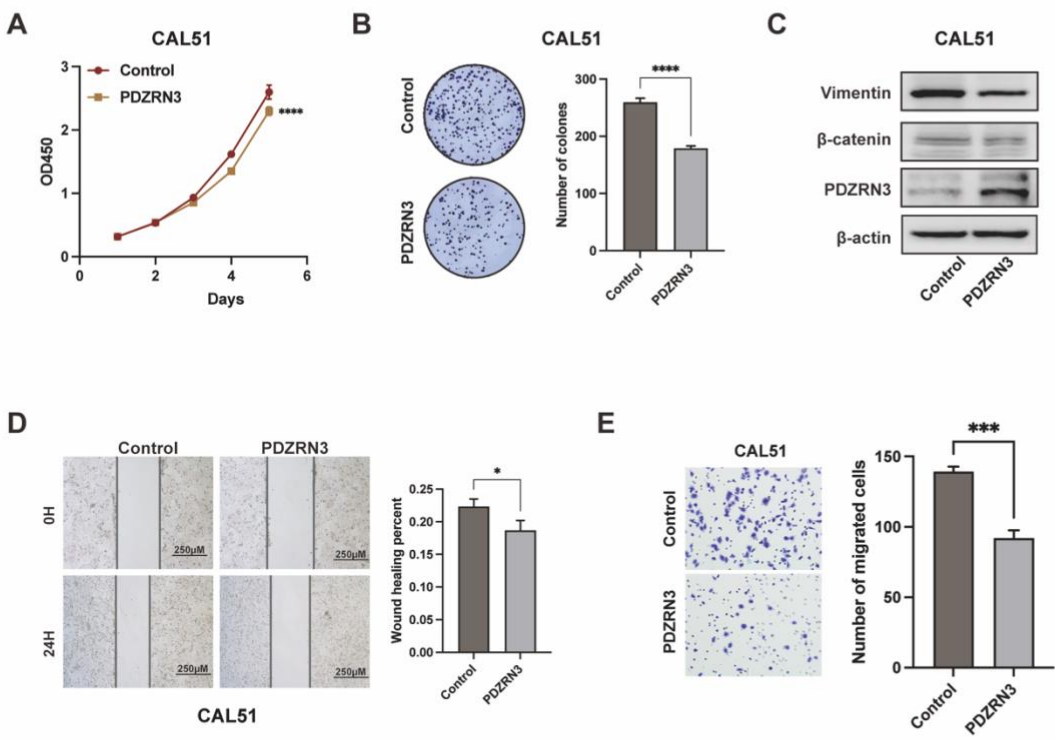

Figure S4 upregulation of PDZRN3 inhibited the proliferation and migration of CAL51 breast cancer cells.

(A) The CCK-8 assay was performed to measure the proliferation capacity of CAL51 cells. (B) The colony formation assay, and corresponding statistical analysis of CAL51 cells. The effect of PDZRN3 on the migration of CAL51 cells determined by transwell (C) and wound healing (D) assays. (E) Western blot analysis revealed that upregulation of PDZRN3 inhibited the cell cycle progression in CAL51 cells.

Table S1 the Antibody information used in this research.

	Antibodies	Sourse	Cat.No.
**Western blot**	β-actin	Cell Signaling Technology	3700
	P-21	Proteintech	10355-1-AP
	P-16	Proteintech	10883-1-AP
	Cyclin B1	Santa Cruz	SC-752
	Cyclin D1	Santa Cruz	SC-753
	GAPDH	Sangene Biotech	KM9002
	FBXL6	Bioss Antibodys	bs-16041R
	PDZRN3	Abcam	ab272628
	β-catenin	Cell Signaling Technology	8480
	Vimentin	Immunoway	YT4880
	Ki67	BD pharmingen	PAC047Ra01

Table S2 Oligonucleotides used for RT-qPCR.

Name	Sequence (5' to 3')
DCAF13 up	GAACTCCTAGCGGACACCT
DCAF13 low	CAACTTGGTTTCGCGGACATA
USP39 up	TTGGAAGAGGCGAGATAA
USP39 low	AGGAGCATCAATCATCATC
PSMD14 up	TCGGAAGCCTAACTACAGCGA
PSMD14 low	ATTATTGAGGTCAACGGCAG
PDZRN3 up	ATTATTGAGGTCAACGGCAG
PDZRN3 low	AGGGCCATGATATGTTCAAAG
TLE3 up	AACCACCATGAACTCGATCAC
TLE3 low	TCACTGTCGTATCGGCTCAAG
SOCS2 up	TTAAAAGAGGCACCAGAAGGAAC
SOCS2 low	AGTCGATCAGATGAACCACACT
SKP2 up	CTTTACTATTAGTGACAAGAGCTGG
SKP2 low	TGGCTGGACTTGAGTTTGGA
FBXL6 up	GGAGACCGCATTCCCTTGG
FBXL6 low	AAAACCGATTGGGCATAAGCC
PSMD14 up	TGTGGAGGCAGTTGATCCAG
PSMD14 low	TCCACACCAGAAAGCCAACA
